# Neointimal hyperplasia after endoluminal injury in mice is dependent on tissue factor- and angiopoietin-2 dependent interferon gamma production by fibrocytes and macrophages

**DOI:** 10.3389/fimmu.2024.1345199

**Published:** 2024-06-07

**Authors:** Daxin Chen, Ke Li, Lin-Lin Wei, Ning Ma, John H. McVey, Anthony Dorling

**Affiliations:** ^1^ Department of Inflammation Biology, School of Immunology and Microbial Sciences, King’s College London, Guy’s Hospital, London, United Kingdom; ^2^ Core Research Laboratory, The Second Affiliated Hospital, Xi’an Jiatong University, Xi’an, China; ^3^ School of Bioscience & Medicine, Faculty of Health and Medical Sciences, University of Surrey, Guildford, United Kingdom

**Keywords:** tissue factor (TF), intimal hyperplasia (IH), thrombin (F2), interferon gama (IFNγ), fibrocyte

## Abstract

**Background:**

The intimal hyperplasia (IH) and vascular remodelling that follows endovascular injury, for instance after post-angioplasty re-stenosis, results in downstream ischaemia and progressive end organ damage. Interferon gamma (IFNγ) is known to play a critical role in this process. In mouse models we have previously shown that fibrocytes expressing tissue factor (TF) are recruited early to the site of injury. Through thrombin generation and protease activated receptor-1 (PAR-1) activation, fibrocytes secrete angiopoietin-2, stimulate neointimal cell proliferation, inhibit apoptosis and induce CXCL-12 production, all of which contribute to the progressive IH that then develops. In this study we investigated the relationship between TF, angiopoietin-2 and IFNγ.

**Methods and results:**

IH developing in carotid arteries of wild-type mice 4 weeks after endoluminal injury contained a significant proportion of IFNγ+ fibrocytes and macrophages, which we show, using a previously defined adoptive transfer model, were derived from circulating CD34+ cells. IH did not develop after injury in IFNγ-deficient mice, except after transplantation of WT bone marrow or adoptive transfer of WT CD34+ cells. *In vitro*, CD34+ cells isolated from post-injury mice did not express IFNγ, but this was induced when provided with FVIIa and FX, and enhanced when prothrombin was also provided: In both cases IFNγ secretion was TF-dependent and mediated mainly through protease activated PAR-1. IFNγ was predominantly expressed by fibrocytes. *In vivo*, all IFNγ+ neointimal cells in WT mice co-expressed angiopoietin-2, as did the small numbers of neointimal cells recruited in IFNγ-/- mice. Adoptively transferred WT CD34+ cells treated with either an anti-TIE-2 antibody, or with siRNA against angiopoetin-2 inhibited the expression of IFNγ and the development of IH.

**Conclusion:**

TF-dependent angiopoietin-2 production by newly recruited fibrocytes, and to a lesser extent macrophages, switches on IFNγ expression, and this is necessary for the IH to develop. These novel findings enhance our understanding of the pathophysiology of IH and expose potential targets for therapeutic intervention.

## 1 Introduction

Intimal hyperplasia (IH) and remodelling of arteries occurs in chronic vascular diseases and underpins multiple and diverse human diseases, including hypertension, post-angioplasty restenosis, and transplant arteriosclerosis (1, 2).

IFNγ is a critical mediator of IH (3). The most prevalent hypothesis is that it induces hyperplasia of α-smooth muscle actin (SMA)+ vascular smooth muscle cells (VSMC) (4), promoting expression of platelet derived growth factor (PDGF) receptors (5) and inducing proliferation directly through PI3-kinase dependent phosphorylation of mammalian target of rapamycin (6). In allogeneic transplant models involving infiltration of recipient T cells and monocytes, the source of IFNγ is obvious, but in mechanical injury models, the initial origin of IFNγ is not established.

We previously showed, using a wire-induced endoluminal injury model (7), that bone marrow (BM)-derived fibrocytes were recruited early to the site of injury (8, 9). In this context, the fibrocytes expressed CD34, CD45, CD31, TIE-2, VEGF-R2, E-selectin and collagen-1 (8, 10) and were mobilised into the peripheral blood quickly after the injury. After purifying these cells from the circulation of injured mice, and adoptively transferring into a secondary host on the day of injury (7, 8, 11), we were able to show that these early fibrocyte recruits have an important role in orchestrating IH. Using a combination of different mouse strains and reagents selectively targeting tissue factor (TF) thrombin, protease activated receptors (PARs) and angiopoietins, we also showed that TF- and thrombin-dependent angiopoietin-2 production by these early fibrocytes was critical for neointima formation, inducing proliferation, inhibiting apoptosis and promoting CXCL-12 secretion, to ensure continued fibrocyte recruitment (9, 12–14).

In other published work, we have defined novel links between TF and thrombin mediated signalling and IFNγ secretion by macrophages (15–17), so in this paper, we investigated how IFNγ was involved in the fibrocyte-dependent IH that follows mechanical endovascular injury. Our data provides further insights into the importance of TF- and thrombin-mediated signalling on myeloid lineage cells and should provide the basis for exploring new therapeutic avenues in translational research.

## 2 Materials and methods

### 2.1 Animals and experimental models

Wild-type (WT) mice (C57BL/6 from Harlan Olac Ltd Bicester, UK), IFNγ^-/-^ mice (kind gift from Simon Clare, Wellcome Trust Sanger Institute, Cambridge, UK), ROSA-enhanced yellow fluorescent protein (EYFP) mice (8) and heterozygous mice expressing a human tissue factor pathway inhibitor (TFPI) fusion protein (18) under control of a CD31 promoter (13), were bred and maintained at King’s College London. All genetically modified animals have been maintained for more than 10 generations on a WT background. All procedures were approved by the UK Home Office.

#### 2.1.1 Wire-induced endoluminal carotid artery injury

25 – 30g mice (n=6 per group) were anesthetized by intraperitoneal injection of 0.1 ml/10g solution (1 ml Hypnorm solution (0.315mg fentanyl/ml and 10mg fluanisone/ml) (VetaPharma Ltd, Leeds, UK), 1 ml Hypnovel solution (5mg Midazolam/ml) (Roche, Garden City, UK) and 2 ml dHO_2_). A dissecting microscope (Stemi SV 6, ZEISS, Germany) was used to perform microsurgery with a 0.015-inch angioplasty guide wire (Cook Incorporated, IN 47404, USA). After placing in the left common carotid via the external carotid artery the wire was withdrawn/reinserted 3 times to destroy the endothelial layer, before the external carotid artery was ligated. In some experiments, animals received 100 ng/g of RPTF243, an affinity purified rabbit polyclonal antibody raised and affinity purified against human tissue factor residues 33 to 275 (legacy numbering 1 to 243 (19)) expressed in *E. col i*(a kind gift of Dr Gordon Vehar) or 80 ng/g anti-human TFPI (Product 4903, American Diagnostica via Enzyme Research Laboratories, Swansea, United Kingdom) or equivalent doses of isotype control antibodies, all given IV in 50 µl saline via tail vein immediately after injury.

#### 2.1.2 BM reconstitution

Long bone donor BM was resuspended in fresh RPMI medium. The recipient mice were irradiated with 12 Gy (1200 rad), injected with 1x10^7^ BM cells and isolated for 4 weeks prior to experimentation.

#### 2.1.3 Adoptive transfer of CD34+ cells

CD34^+^ cells were purified from the blood of mice 2-4 days after injury. Blood was collected into 1mM EDTA or 3.2% sodium citrate and diluted with 2% foetal calf serum (FCS) in phosphate buffered saline (PBS). After spinning, cells were re-suspended in Ammonium Chloride Potassium (ACK) buffer, incubated at room temperature (RT) for 25 minutes, then washed to remove platelets. CD34^+^ cells with an average purity of 95% were purified using magnetic beads (Miltentyi Biotech, Surrey UK) (8, 9). 1x10^6^ CD34^+^ cells, isolated from 3-4 donor mice, were adoptively transferred, via a tail vein, to a second mouse on the day of injury. In some experiments, prior to transfer, CD34+ cells were first incubated with 100µg/ml rat anti-mouse TF antibody (20),or 10µg/ml anti-mouse TIE2 antibody (Abcam, Cambridge, UK) and equal dose of isotype control antibodies, or total of 250 pmols siRNA (see below) for 1 hour in a 24-well plate.

### 2.2 Morphometric analysis and immunohistology

Vessels were embedded in optimum cutting temperature compound (OCT) (VWR International, Dorset, UK) before cross-sectioning and staining with Accustain Elastin Stain kit (Sigma). Morphometric analysis was done on an Olympus U-ULH microscope (Olympus Optical Co Ltd, Tokyo, Japan). Areas were determined with Image-Pro Plus TM software version 4.0 (Media Cybernetics, Silver Spring, MD, USA). Three random sections from each of six wire-injured arteries were examined by an investigator blinded to the identity of the sections. The mean value from each vessel was used to prepare figures and statistics.

For immunofluorescence (IF) analysis, OCT-embedded vessels were cut into 5 µm sections prior to fixing in methanol for 60 minutes at –20°C. After immersion for 30 minutes in 1% BSA (Sigma)-PBS, frozen sections were incubated overnight at 4°C with combinations of the following antibodies: rabbit polyclonal collagen 1, rat anti-mouse F4/80, rabbit polyclonal F4/80, rat anti-mouse collagen 1 and hamster anti-mouse IFNγR (all from Abcam), mouse anti-human αSMA (Sigma), rat anti-mouse monoclonal IFNγ (Invitrogen), goat anti-angiopoietin-2 (Santa Cruz biotechnology Inc.), and rabbit anti-mouse TF (American Diagnostica.inc. USA). All stained sections were mounted in Vectashield medium with DAPI (Vector Laboratories). Sections were analysed by a Leica DM-IRBE confocal microscope (Leica, Wetzlar, Germany) equipped with Leica digital camera AG and a confocal laser scanning system with excitation lines at 405, 488, 543, and 560 nm at magnifications 10x/0.40CS and 20x/0.70IMM (Leica, Planapo, Wetzlar, Germany). Images were processed using the Leica-TCS-NT software associated with the Leica confocal microscope. Three random sections from each of six wire-injured arteries were examined by an investigator blinded to the identity of the sections. The mean value from each vessel was used to prepare figures and statistics. Isotype-matched antibodies were used in initial sections to confirm the specificity of staining of individual antibodies (data not shown).

### 2.3 FXa &thrombin generation and IFNγ secretion

CD34+ cells (2x10^4^ cells per well in a 96-well plate) were washed and suspended in 200μl Dulbecco modified Eagle medium (DMEM; Sigma-Aldrich MO, USA) containing 0-320 nM human Factor X (FX) and 0- 20 nM human factor VIIa (FVIIa) at 37°C. In some experiments, cells were first incubated with 100μg/ml of rat anti-mouse TF (20). After 20 minutes, aliquots or the reaction mixture were transferred into Tris-EDTA buffer with the chromogenic substrate S-2222 (Chromagenix, Milan, Italy) to assess FXa generation. Absorbance at 405 nm was converted to give the FXa concentration after comparison to defined standard controls. A similar assay was used to assess thrombin generation, using either 10 nM FX and 6 nM FVIIa, OR 10nM FXa, with added pre-prepared 6 nM human factor Va (FVa) and 0-10nM human prothrombin (all from Enzyme Research Laboratories) added in HEPES-buffered saline (Life Technologies, Grand Island, NY). At defined times, aliquots of the reaction mixture were transferred into Tris-EDTA buffer with the chromogenic substrate S-2238 (Chromagenix, Milan, Italy) to assess thrombin generation as for FXa, using absorbance at 405 nm and standard controls to calculate thrombin concentration.

For IFNγ expression, 2x10^4^ CD34^+^ cells were seeded on a round glass coverslip in a 24-well plate and serum starved for 24 hours, before addition of varying concentrations of either FX and FVII, FXa, FVIIa, FX and prothrombin or thrombin (0-100nM) (Enzyme Research Laboratories). The cells were incubated for 5 days in Iscove modified Dulbecco medium (IMDM; Sigma-Aldrich, MO, USA) supplemented with 2% FCS (StemCell Technology, Grenoble, France). IFNγ secretion was measured by ELISA (following manufacturer’s instructions (R&D Systems, Abingdon, UK)). The same kit was used to measure plasma concentrations.

PAR-induced cell signalling was measured in serum starved cells treated for 30 minutes with 0 - 80 µM of either PAR-1 (FLLRN), PAR-2 (FSLLRY-amide) or PAR-4 (Trans-cinnamoyl YPGKF-NH2) antagonists (Peptides International, Louisville, KY) before the cells were stimulated by FVIIa+FX or FVIIa+FX+FII at indicated doses. Alternatively, cells were stimulated with 0 - 100 µM of PAR-1 (SFLLR-amide), PAR-2 (2-Furoyl-LIGRLO-Amide) or PAR-4 (GYPGKF) agonists (Peptides International) at the indicated doses. In some assays, cells were first treated with siRNA for angiopoietin 2 or the fluorescein conjugated control siRNA, using 2 x 10^5^ CD34+ cells per well were seeded in a six well tissue culture plate (see below).

### 2.4 siRNA transfection

50 pmols siRNA for angiopoietin 2 (sc-39294) and fluorescein conjugated controls (sc-36869) (Santa Cruz biotechnology Inc. Texas 75220, USA) were used to transiently transfect 2 x 10^5^ CD34+ cells per well in a six-well plate using a siRNA transfection system supplied by Santa Cruz Biotechnology Inc. 24 hours later, cells were washed twice in serum-free medium and some were then incubated with 10nM thrombin for further incubation with the culture medium containing 2% FCS. Analysis of cell phenotype or supernatants was performed 48 hours later. For *in vivo* use, 5 wells of transfected cells were combined to treat a single mouse.

### 2.5 Immunocytochemistry

CD34+ cells spread onto a glass coverslip (VWR International, Leuven, Belgium) were fixed for 10 minutes with methanol at –20°C, before incubation at RT for 60 minutes with one or more of the following antibodies: mouse anti–human α-SMA conjugated with Cy3 (Sigma-Aldrich, St Louis, MO), rat anti-mouse monoclonal IFNγ (Invitrogen), rat anti-mouse CD31 (BD) and rabbit anti- collagen-1, rabbit anti-Angiopoietin-2, and rabbit anti-TIE-2 (all from Abcam). Second layer staining was with a goat anti-rabbit IgG-FITC, goat anti-rat IgG-FITC or a rabbit anti-goat IgG-FITC (all from Sigma-Aldrich). Stained cells were mounted in Vectashield medium with DAPI (Vector Laboratories), and analysed by a Leica DMIRBE confocal microscope (Leica, Wetzlar, Germany) as above.

### 2.6 Statistical analysis

Statistical analysis was performed with GraphPad Prism software. Mann-Whitney or T test was used for comparison of 2 groups and the Kruskal-Wallis test for ≥3 groups. All data were reported as mean ± SEM. A P value of <0.05 was considered statistically significant.

## 3 Results

### 3.1 IFNγ secretion by early neointimal myeloid cells is required for IH

**Figure 1:**
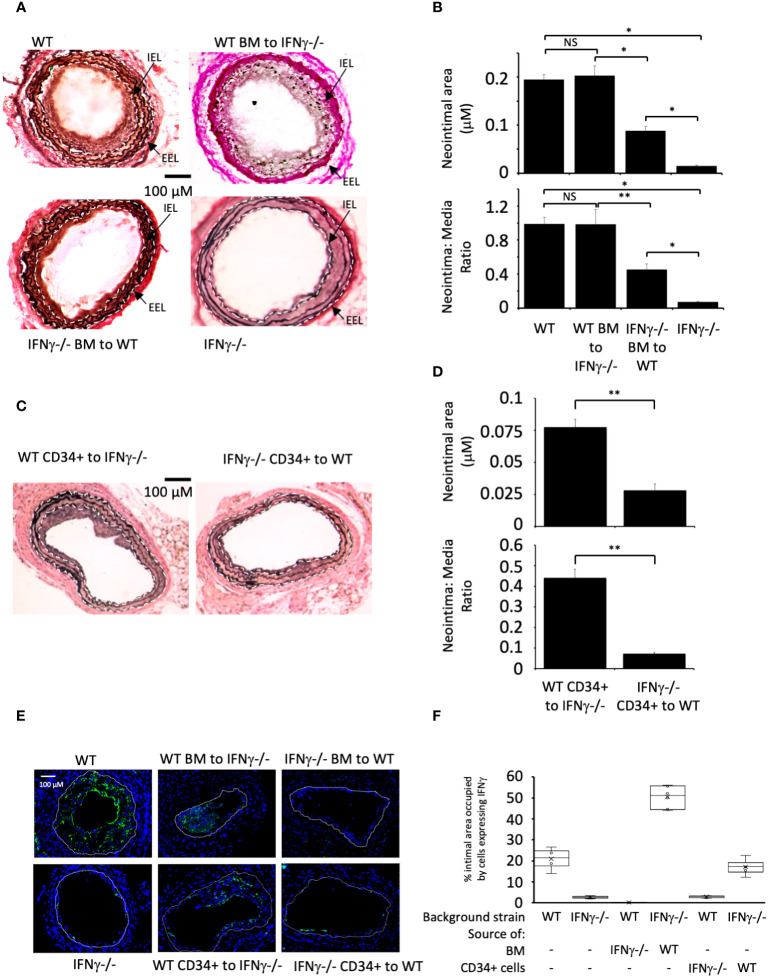
IFNγ secretion by BM-derived CD34+ cells is required for IH. **(A, C)** Cross sectional images of carotid arteries 28 days post-injury stained with Accustain. A compares WT and IFNγ-/- animals and groups that underwent reciprocal BM transplantation as indicated. C compares animals that, on the day of injury, received CD34+ cells isolated 2-4 days post-injury from third-party mice as indicated. Internal elastic lamina (IEL) and external elastic lamina (EEL) are annotated with white dashed lines and indicated by arrows where appropriate. **(B, D)** Neointimal area (top panel) and intima:media ratio (bottom panel) of vessels taken from animals 28 days post-injury. **(B)** shows animals represented in **(A)**. **(D)** shows animals represented in **(C)**. Data derived from examination of 3 random sections from 6 different vessels. NS, not significant: * p<0.005: ** p<0.002. **(E)** Panels show immunohistology of representative sections through injured mouse carotid arteries harvested on day 28 post-injury. All Sections stained with DAPI (4,6 diamidino-2-phenylindole) nuclear stain (blue) and (green) anti-IFNγ. The annotated white line defines the junction between neointima and media. 3 random sections from each of six arteries, with the investigator blinded to the identity of the animals, were examined. **(F)** Quantitative analysis of the expression of IFNγ in the respective sections shown in E, expressed as the proportion of the intimal area occupied by cells expressing the cytokine. Graphs show box plots with median and interquartile range (IQR) with whiskers showing upper and lower limits and outliers indicated as single data points. Means are represented with ‘x’. Data derived from mean values from 3 random sections taken from 6 different vessels. Measurements taken by an investigator blinded to the identity of the sections. WT, wild-type (C57BL/6); BM, bone marrow; EEL, external elastic lamina; IEL, internal elastic lamina; IFNγ^-/-^, interferon-gamma deficient mice.

Wire induced injury is characterised by immediate loss of endothelium (21), followed by platelet deposition (21, 22), non-occlusive luminal thrombosis (23) and the rapid induction of TF expression by medial VSMC (24). Compared to WT BL/6 mice, IFNγ^-/-^ mice developed little IH when examined 4 weeks post endoluminal injury (**Figure 1A**).

Reciprocal BM reconstitution showed that IFNγ^-/-^ mice reconstituted with WT BM developed IH to same degree as WT, whereas in WT mice reconstituted with IFNγ^–/-^ BM the IH that developed was significantly reduced compared to WT controls (**Figures 1A, B**). Plasma levels of IFNγ reflected the source of BM cells (**Table 1**). These data suggest that IFNγ production is by BM-derived cells and is both necessary and sufficient to mediate IH.

**Table 1:** Serum concentrations of IFNγ 5 days post-wire-induced injury.

Mouse Strain	Treatment	Mean [IFNγ] (pg/ml)	SEM	P value
WT	Recipient of IFNγ-/- BM	3.76	0.74	
IFNγ^-/-^	Recipient of WT BM	16.7	1.5	P=0.03
WT	none	22.16	5.6	
WT	Recipient of IFNγ-/- CD34+ cells	6.21	2.0	
IFNγ^-/-^	Recipient of WT cells	16.1	5.0	
IFNγ^-/-^	none	0	0	P=0.001
WT	Isotype control Ab	19.47	3.4	
WT	Anti-TF Ab	6.59	1.7	
CD31-TFPI-Tg	Isotype control Ab	4.32	1.7	
CD31-TFPI-Tg	Anti-TFPI Ab	13.79	1.6	P=0.002
WT	EYFP CD34+ cells incubated with isotype control Ab	20.76	1.49	
WT	EYFP CD34+ cells incubated with anti-TIE-2 Ab	4.9	0.73	P=0.003
WT	EYFP CD34+ cells incubated with control siRNA	21.98	2.0	
WT	EYFP CD34+ cells incubated with Ang-2 siRNA	5.37	1.23	P=0.003

We have previously defined and validated an adoptive transfer model, in which CD34 + cells injected intravenously at the time of vascular injury outcompete endogenously circulating CD34+ cells for uptake at the site of injury (14). The CD34+ cells are obtained from mice that undergo wire-injury 2-4 days earlier, so reflect cells mobilised into the circulation by the injury. Transfer of IFNγ^-/-^ CD34+ cells into WT mice significantly impaired the development of IH, whereas transfer of WT cells into injured IFNγ^-/-^ mice was associated with development of IH, although not to the extent see in WT mice (**Figures 1C, D**). As above, plasma levels of IFNγ reflected the source of CD34+ cells (**Table 1**). These data suggest that CD34+ cells capable of making IFNγ and recruited early after injury contribute to the development of IH.

Immunofluorescence of sectioned arteries from both BM reconstitution and CD34+ transfer experiments confirmed that when intimal cells came from WT, a substantial proportion of them made IFNγ, whereas when intimal cells came from IFNγ-/- mice, the proportion expressing IFNγ was substantially diminished (**Figures 1E, F**). Staining for IFNγ seen in the IFNγ-/- mice was regarded as artefact. All together, these data imply that IFNγ expression by BM-derived, CD34+ cells recruited to the site of injury makes a significant contribution to the subsequent development of IH.

**Figure 2:**
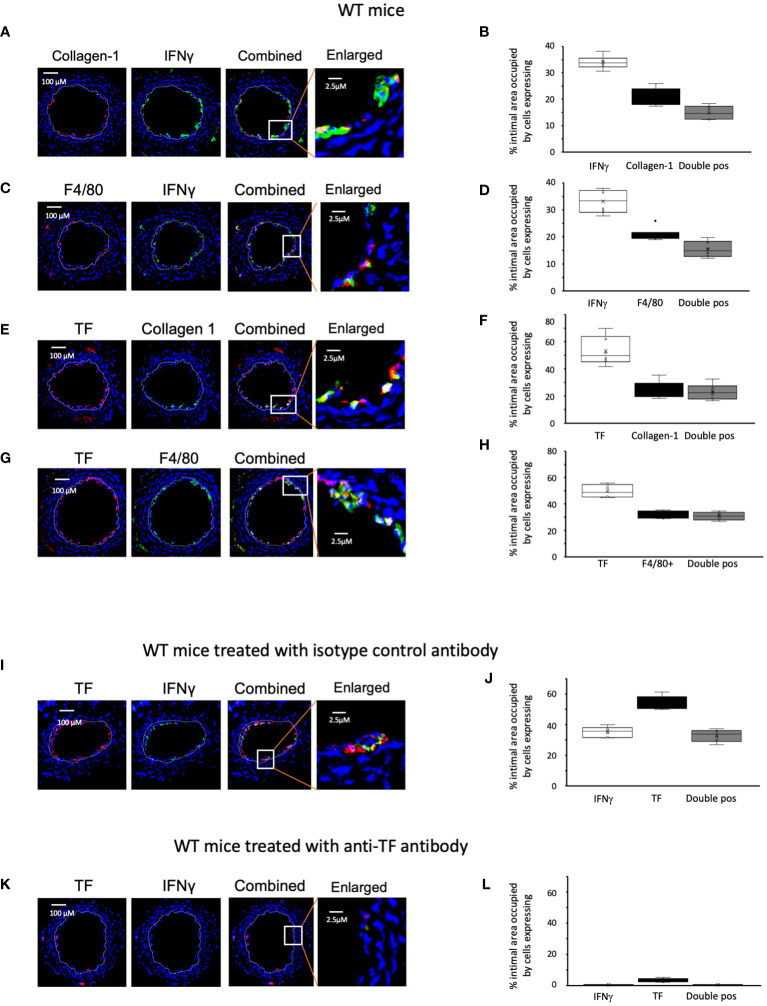
Phenotype of early WT neointimal cells expressing IFNγ **(A, C, E, G, I, K)** Panels show immunohistology of representative sections through injured WT mouse carotid arteries harvested on day 5 post-injury. **(A–G)** are from untreated mice. I and K were treated with control or anti-TF antibody on day of injury as indicated. All sections stained with DAPI (4,6 diamidino-2-phenylindole) nuclear stain (blue) and (red) anti-collagen-1, anti-F4/80 or anti-TF as indicated, plus (green) anti-IFNγ, anti-collagen-1 or anti-F4/80 as indicated. The annotated white line defines the junction between neointima and media. The enlarged image shows the portion of artery indicated by the white box. **(B, D, F, H, J, L)** Quantitative analysis of the expression of the markers shown in the respective sections, expressed as the proportion of the intimal area occupied by cells expressing a particular marker(s) as indicted in each panel. Graphs show box plots with median and interquartile range (IQR) with whiskers showing upper and lower limits and outliers indicated as single data points. Means are represented with ‘x’. Data derived from mean values from 3 random sections taken from 6 different vessels. Measurements taken by an investigator blinded to the identity of the sections.

We examined the phenotype of neointimal cells expressing IFNγ on day 5 post-injury, by which time the acute thrombotic changes associated with injury have cleared (9). Approximately 35% of the intimal area was occupied by cells expressing IFNγ (**Figures 2A–D**). Collagen type 1+ and F4/80+ cells each occupied 20-30% of intimal area (**Figures 2A–H**). Equivalent proportions of the intimal area were occupied by cells co-expressing IFNγ with collagen-1 (median 14% [IQR 12.6-18], **Figures 2A, B**), and cells co-expressing IFNγ with F4/80 (median 15% [IQR 13.6-18], **Figures 2C, D**). Since collagen-1 and F4/80 appeared to be co-expressed by cells occupying approximately 5% of the intimal area (**Supplementary Figure S1**), we conclude that 25% of the intimal area was occupied by myeloid cells expressing IFNγ and that >70% of the cells expressing IFNγ were therefore myeloid lineage cells.

### 3.2 IFNγ secretion by early neointimal myeloid cells is dependent on TF and coagulation protease generation

We have previously shown that development of IH in this model is TF dependent. We confirmed that virtually all collagen-1+ and all F4/80+ cells co-expressed TF (**Figures 2E–H**). In WT controls treated with an isotype matched antibody at the time of injury (**Figures 2I, J**), by day 5 a median of 36% [IQR 33-38] of intimal cells expressed IFNγ. In contrast, in mice treated with an anti-TF antibody (**Figures 2K, L**) this was significantly reduced (median 0.4% [IQR 0.38-0.7] p=0.004) and plasma IFNγ levels were significantly reduced in these mice (**Table 1**). These data suggested that both IFNγ expression by intimal cells and plasma levels of IFNγ were dependent on TF expression.

**Figure 3:**
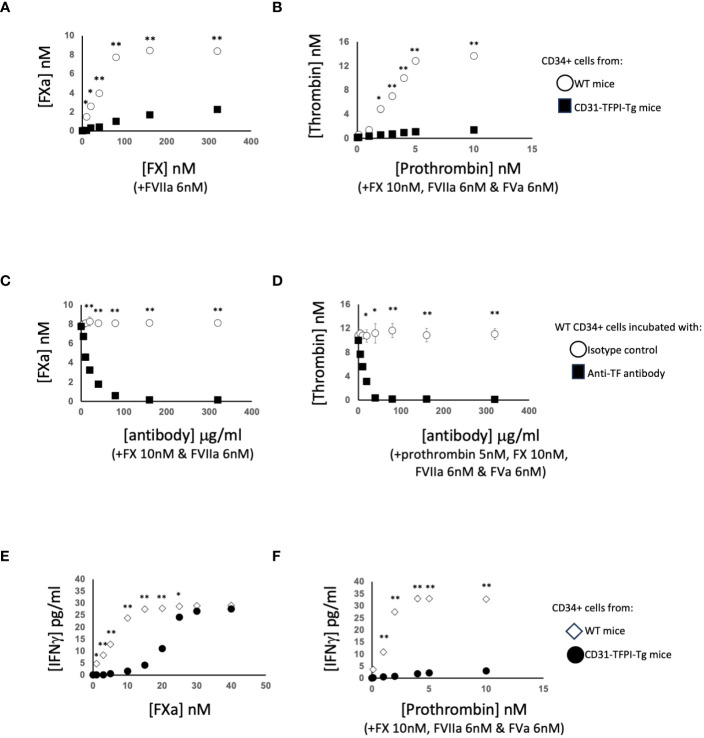
Generation of coagulation proteases and IFNγ by CD34+ cells. CD34 cells purified from the blood of mice 2-4 days post-wire-induced injury. **(A, C)** FXa generation, assessed by conversion of chromogenic substrate S-2222, in presence of FVIIa (6nM) after **(A)**: addition of increasing concentrations of FX or **(C)** presence of FX (10nM) followed by titration of increasing concentrations of control or inhibitory rat anti-TF antibody as indicated. **(B, D)** thrombin generation, assessed by conversion of chromogenic substrate S-2238 in presence of FX (10nM), FVIIa (6nM) and FVa (6nM) after **(B)** addition of increasing concentrations of prothrombin or **(D)** presence of prothrombin (5nM) followed by titration of increasing concentrations of control or inhibitory anti-TF antibody. In **(A, B)** black squares – CD31-TFPI-Tg CD34+ cells: white circles WT CD34+ cells. In **(C, D)** black squares – WT cells with anti-TF: white circles WT cells with isotype control antibody. **(E, F)** IFNγ secretion after 5 days incubation of WT CD34+ cells (white diamonds) or CD31-TFPI-Tg cells (black circles) with **(E)** increasing concentration of FXa as indicated or **(F)** increasing concentrations of prothrombin as indicated in presence of FX (10nM), FVIIa (6nM) and FVa (6nM). In all panels, *p<0.01: ** p<0.001.

To interrogate these data in more detail, we performed several rounds of additional experiments. First, we purified CD34+ cells from the blood of WT mice 2-4 days post-injury. We confirmed these cells expressed TF, as they could convert FX to FXa in a dose-dependent way when FVIIa was present (**Figure 3A**), and prothrombin to thrombin when factors FVIIa, FX and FV were present (**Figure 3B**) and both these were inhibited by an anti-TF antibody (**Figures 3C, D**).

**Figure 4:**
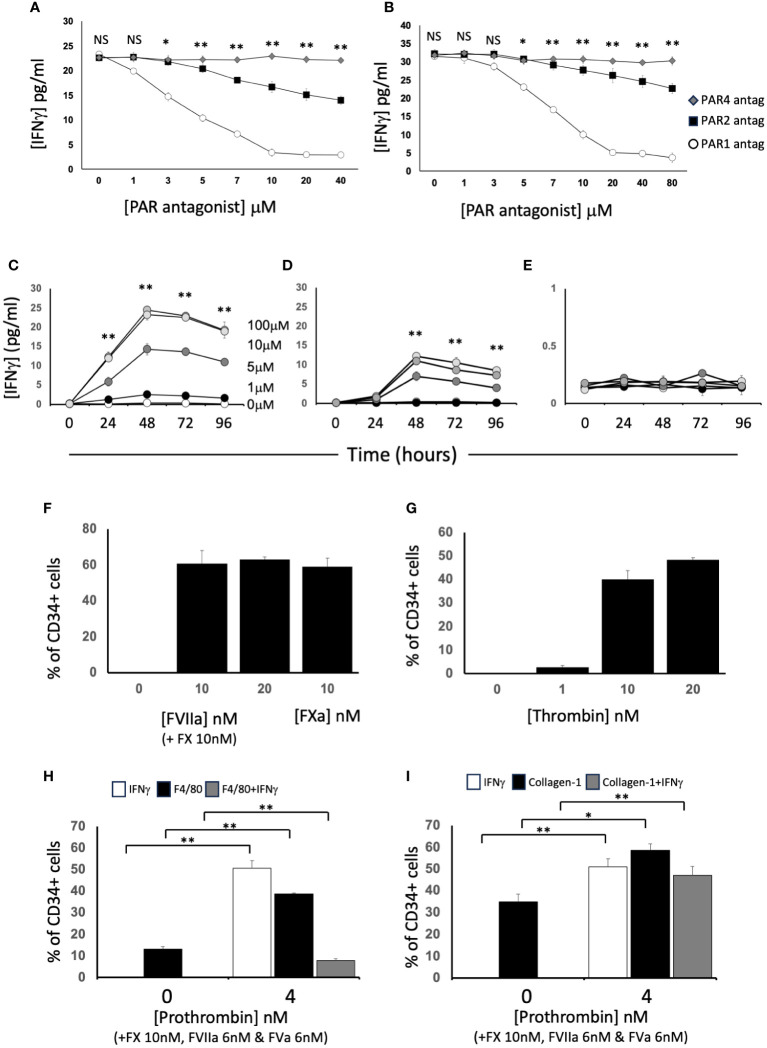
Dissection of IFNγ expression by CD34+ cells. CD34 cells purified from the blood of mice 2-4 days post-wire-induced injury. **(A, B)** IFNγ secretion after 5 days incubation of WT CD34+ cells with **(A)** FXa (10nM) or **(B)** thrombin (10nM) in presence of increasing concentrations of antagonists for PAR-1 (white circles). PAR-2 (black square) or PAR-4 (grey diamonds). **(C–E)** IFNγ secretion after 1-4 days incubation of WT CD34+ cells with **(C)** PAR-1 agonist; **(D)** PAR-2 agonist or **(E)** PAR-4 agonist at 0-100μM as indicated. *p<0.01: ** p<0.001. **(F-I)** Immunocytochemical analysis of CD34+ cells stained with anti- IFNγ after stimulation with **(F)** FX (10nM) and various concentration of FVIIa as indicated or FXa (10nM); **(G)** increasing concentrations of thrombin; **(H, I)** FX (10nM), FVIIa (6nM) and FVa (6nM) with prothrombin at the concentrations indicated. In **(H)**, cells also stained with ant-F4/80 and in **(I)** with anti-collagen-1. Data derived from examination of 3 cover slips from at least 2 different experiments. In **(H, I)** white bars show the total proportion of cells expressing IFNγ; black bars show total proportion expressing F4/80 **(H)** or collagen-1 **(I)** grey bars show proportion of cells co-expressing both. *p<0.01; **p<0.001. NS, not significant.

After incubation with FXa +/-prothrombin, IFNγ was released into the medium (**Figures 3E, F**) dose-dependently. IFNγ secretion was partially inhibited by titration of a PAR-2 antagonist, and almost completely inhibited by a PAR-1 (but not PAR-4) antagonist (**Figures 4A, B**), suggesting that both FXa and thrombin acted primarily through PAR-1. In support of this a PAR-1 agonist induced the secretion of twice the amount of IFNγ in a dose and time dependent manner compared to a PAR-2 agonist (**Figures 4C, D**). A PAR-4 agonist induced no IFNγ secretion (**Figure 4E**). At baseline, none of the CD34+ cells expressed IFNγ (**Figures 4F, G**), but it was expressed by 50-60% after incubation with either FXa or thrombin. In addition, in this *in vitro* system, these manipulations initiated expansion of both F4/80+ cells (**Figure 4H**), from 12 to 40% of the CD34+ cells and collagen-1+ cells (**Figure 4I**), from 35 to 60%. However, the vast majority of IFNγ+ cells were collagen-1+, with <10% co-expressing F4/80. These data indicate that TF on the surface of CD34+ cells, via FXa and thrombin generation, induces IFNγ expression and secretion primarily from collagen-1+ cells, via activation of PAR-1 and to a lesser extent PAR-2.

**Figure 5:**
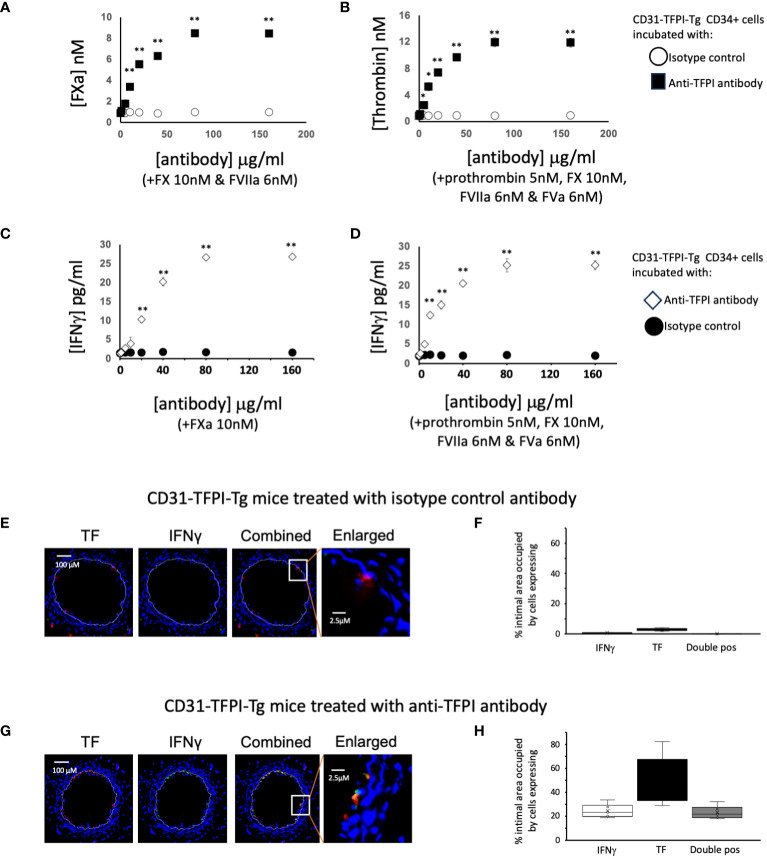
To illustrate the importance of TFPI expression in CD31-TFPI-Tg mice. **(A–D)** Generation of coagulation proteases and IFNγ by CD34+ cells from CD31-TFPI-Tg mice. CD34 cells purified from the blood of mice 2-4 days post-wire-induced injury. **(A)** FXa generation, assessed by conversion of chromogenic substrate S-2222, in presence of FX (10nM) and FVIIa (6nM) after titration of increasing concentrations of control (white squares) or inhibitory anti-TFPI antibody (black squares). **(B)** thrombin generation, assessed by conversion of chromogenic substrate S-2238 in presence of prothrombin (5nM), FX (10nM), FVIIa (6nM) and FVa (6nM) after titration of increasing concentrations of control (white circles) or inhibitory anti-TFPI antibody (black squares). **(C, D)** IFNγ secretion after 5 days incubation of in presence of increasing concentrations of inhibitory anti-TFPI antibody (white diamonds) or isotype control (black circles) after stimulation with **(C)** FXa (10nM) or **(D)** prothrombin (5nM) with FX (10nM), FVIIa (6nM) and FVa (6nM). *p<0.01: ** p<0.001. **(E–H)** Phenotype of early neointimal cells expressing IFNγ, after treatment, on day of injury, with either an isotype control antibody **(E, F)** of inhibitory anti-TFPI antibody **(G, H)**. **(E, G)** Panels show immunohistology of representative sections through injured mouse carotid arteries harvested on day 5 post-injury. All Sections stained with DAPI (4,6 diamidino-2-phenylindole) nuclear stain (blue) and (red) anti-TF, plus (green) anti-IFNγ, as indicated. The annotated white line defines the junction between neointima and media. The enlarged image shows the portion of artery indicated by the white box. **(F, H)** Quantitative analysis of the expression of the markers shown in the respective sections, expressed as the proportion of the intimal area occupied by cells expressing a particular marker(s) as indicted in each panel. Graphs show box plots with median and interquartile range (IQR) with whiskers showing upper and lower limits and outliers indicated as single data points. Means are represented with ‘x’. Data derived from mean values from 3 random sections taken from 6 different vessels. Measurements taken by an investigator blinded to the identity of the sections.

Next we studied CD34+ cells from CD31-TFPI-Tg mice (13). In the peripheral blood of these mice, circulating CD34+ cells expressing CD31+ co-express a TFPI fusion protein (9, 11, 14). Compared to cells from WT mice, cells from CD31-TFPI-Tg were unable to convert FX to FXa in a dose-dependent manner when FVIIa was present (**Figure 3A**), unless an inhibitory anti-TFPI antibody was titrated in (**Figure 5A**). In accordance, they were unable to convert prothrombin to thrombin when factors FVIIa, FX and FV were present (**Figure 3B**), unless the inhibitory anti-TFPI antibody was present (**Figure 5B**). Incubation with FXa with or without prothrombin failed to induce IFNγ secretion at the same concentrations that worked in WT (**Figures 3E, F**) except when the anti-TFPI antibody was present (**Figures 5C, D**). These data confirm that inhibition of TF on only the fraction of CD31+ myeloid cells, which are predominantly fibrocytes (9, 11, 14), by the transgenic fusion TFPI fusion protein inhibited IFNγ secretion.

Finally we looked at the early intima in wire-injured CD31-TFPI-Tg mice which, as we have previously described, fail to develop progressive IH. There were few detectable IFNγ+ cells in the day 5 intima (**Figures 5E, F**) of mice treated with an isotype control antibody (median 0.6% [IQR 0.5-0.9] and circulating levels of IFNγ were low (**Table 1**). However, if these animals were treated with the inhibitory anti-hTFPI antibody at the time of injury (**Figures 5G, H**), the proportion of IFNγ+ cells increased significantly (median 23% [IQR 21-27] p=0.004), all of which were TF+ and plasma IFNγ levels approached those seen in WT animals (**Table 1**).

All these data suggest that intimal IFNγ expressed by newly recruited CD34+ cells is TF-dependent. Moreover, the TF expressed by fibrocytes is primarily responsible for FXa and thrombin generation and these two enzymes stimulate IFNγ release by these cells predominantly through activation of PAR-1, with PAR-2 playing a lesser role.

### 3.3 IFNγ secretion by early neointimal myeloid cells is dependent on angiopoietin-2 secretion

**Figure 6:**
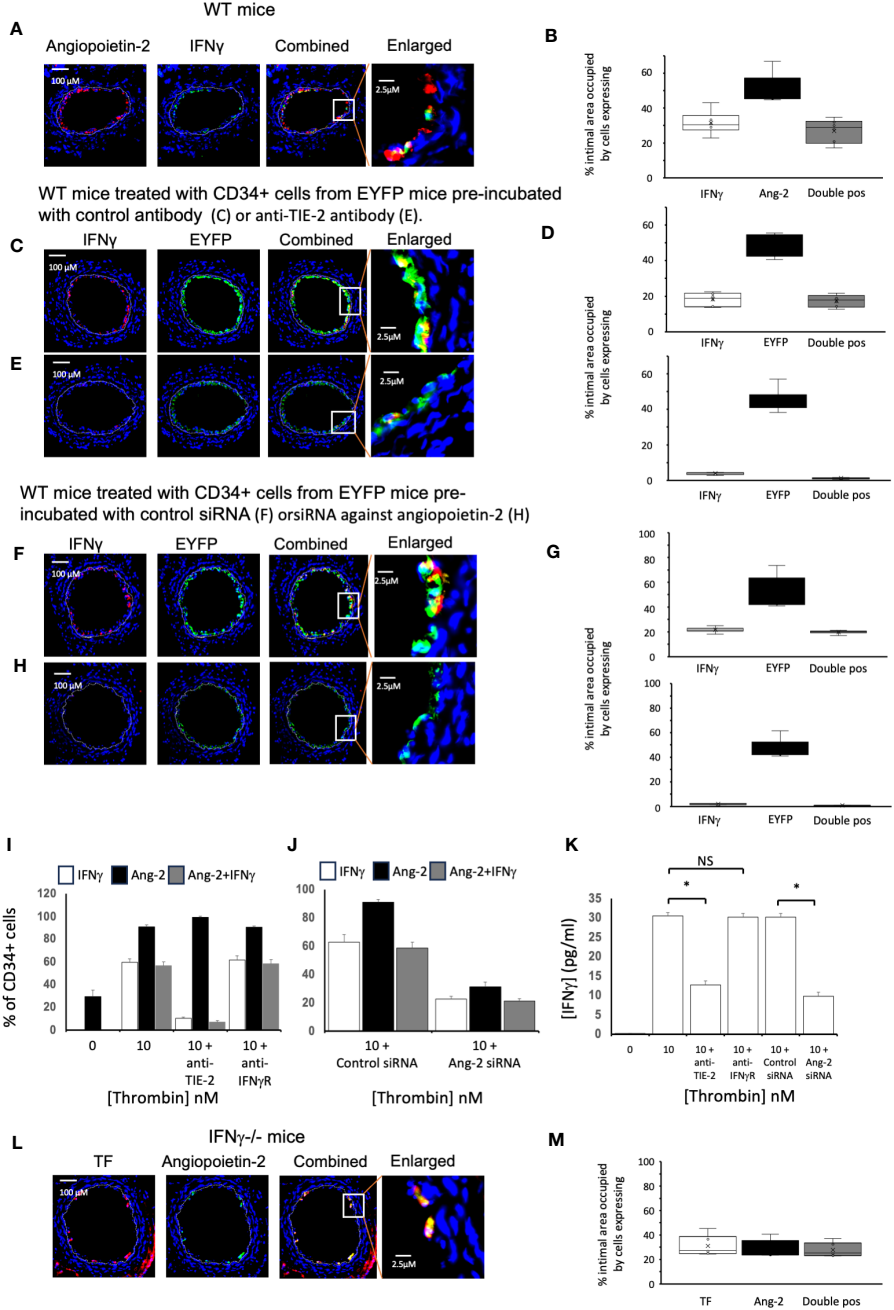
Links between angiopoietin-2 and IFNγ Different strains of mice, as indicated, were studied after wire induced injury +/- treatment with CD34+ cells. CD34 cells purified from the blood of mice 2-4 days post-wire-induced injury. **(A, C, E, F, H, L)** Panels show immunohistology of representative sections through injured mouse carotid arteries harvested on day 5 post-injury. Sections stained with DAPI (4,6 diamidino-2-phenylindole) nuclear stain (blue) and (red) anti-Angiopoietin-2, anti- IFNγ or anti-TF as indicated, plus (green) anti-IFNγ or anti-angiopoietin-2 as indicated. The annotated white line defines the junction between neointima and media. **(A)** WT mice. **(C, E, F, H)** WT mice treated with CD34+ cells from EYFP mice, which spontaneously fluoresce as shown. The transferred CD34+ cells were pre-treated with **(C)** Isotype control antibody: **(E)** anti-TIE-2 antibody: **(F)** control siRNA: **(H)** siRNA against angiopoietin-2. **(L)** IFNγ^-/-^ mice. The enlarged image shows the portion of artery indicated by the white box. **(B, D, G, M)** Quantitative analysis of the expression of the markers shown in the respective sections, expressed as the proportion of the intimal area occupied by cells expressing a particular marker(s) as indicted in each panel. Graphs show box plots with median and interquartile range (IQR) with whiskers showing upper and lower limits and outliers indicated as single data points. Means are represented with ‘x’. Data derived from mean values from 3 random sections taken from 6 different vessels. Measurements taken by an investigator blinded to the identity of the sections. **(I, J)** Quantification of immunocytochemical analysis of the proportion of CD34+ cells stained with anti-IFNγ (white bars) and anti-angiopoietin-2 (black bars) after stimulation with thrombin at the concentrations indicated for 5 days. CD34 cells purified from the blood of mice 2-4 days post-wire-induced injury. Grey bars indicate the proportion of cells expressing both. In I, cells pre-incubated with inhibitory anti-TIE-2 or anti- IFNγR antibodies. In **(J)** cells pre-treated with control or siRNA targeted against angiopoietin-2. Data derived from examination of 3 cover slips from at least 2 different experiments. **(K)** IFNγ secretion from the WT CD34+ cells incubated in the conditions illustrated in **(I, J)**. *p<0.01: NS, not significant.

We have previously shown that angiopoietin-2 secretion by recruited myofibrocytes is necessary for the development of IH, by inducing myofibrocyte proliferation through TIE-2, and promoting CXCL-12 secretion to mediate continuous recruitment of new myofibrocytes from the circulation as well as through induction of angiopoietin-2 expression by non-myofibrocytes (14). In day 5 sections from WT mice, more than 90% of the cells expressing IFNγ co-expressed angiopoietin-2 (**Figures 6A, B**) although only approximately half of the angiopoietin-2 positive cells were IFNγ+.

We adoptively transferred CD34+ cells from ROSA-EYFP mice, which express yellow fluorescent protein in all cells. By day 5, 35% of the cells in the new intima expressed IFNγ and all IFNγ+ cells were EYFP+ (**Figures 6C, D**), indicating that in the adoptive transfer model, all of the early intimal IFNγ+ cells come from cells injected into the circulation. Incubation with either an inhibitory anti-TIE-2 antibody prior to transfer (**Figures 6C–E**) or with siRNA targeted to angiopoietin-2 (**Figures 6F–H**), significantly reduced the intimal area occupied by IFNγ+ cells, compared to controls (in both cases from approx. 20% to <5%, in both cases p=0.004), without influencing the overall recruitment of EYFP+ cells, and substantially reduced levels of plasma IFNγ (**Table 1**). Thus, inhibition of angiopoietin-2 production by, or signalling through TIE-2 on the recruited CD34+ cells, significantly inhibits the expression of IFNγ by these early intimal cells.

Experiments *in vitro* with WT CD34+ cells suggested that although approximately 30% of the CD34+ cells isolated on day 3 post injury expressed angiopoietin-2+, none expressed IFNγ (**Figure 6I**). However, after stimulation with thrombin, approximately 60% of cells were IFNγ + (**Figure 6I**), almost all of which co-expressed angiopoietin-2. This was associated with detectable IFNγ in the supernatant (**Figure 6K**). Incubation with an anti-TIE-2 antibody significantly reduced the proportion of IFNγ+ cells (**Figure 6I**) and inhibited IFNγ secretion (**Figure 6K**) without altering the expression of angiopoietin-2. Similar results were seen when the cells were incubated with siRNA against angiopoietin-2 (**Figures 6J, K**), though this was achieved with significant reduction in the expression of angiopoietin-2 (**Figure 6J**). In contrast, inclusion of an inhibitory antibody against IFNγR had no impact on the expression of either angiopoietin-2 or IFNγ (**Figure 6I**). Thus, thrombin induced angiopoietin-2 expression induces IFNγ expression and secretion.

Finally, we confirmed that in day 5 sections from injured IFNγ^-/-^ mice approximately 25% of the intima was occupied by angiopoietin-2+ cells, almost all of which were TF+. Thus, expression of angiopoietin-2 is upstream of IFNγ (**Figures 6L, M**).

All these data suggest that the angiopoietin-2 secretion induced in CD34+ cells circulating post injury by TF-mediated FXa and thrombin is directly involved in the induction of IFNγ by intimal myeloid cells and that this is critical for the development of IH.

## 4 Discussion

IH following vascular injury occurs because of a progressive accumulation of intimal cells expressing smooth muscle actin (SMA), which combined with vascular remodelling causes stenosis and downstream ischaemia. The importance of IFNγ in this process has been recognized for some time. For instance, rats treated to increase levels of IFNγ inhibitory proteins developed significantly reduced neointima formation days 7-14 after balloon injury of carotid arteries (25) and IFNγ-deficient mice showed significantly reduced neointima after endoluminal injury (26). IFNγ is also important for IH developing after other types of injury (27) and exogenously administered IFNγ can mediate IH in transplanted arteries in the absence of host leukocytes (5, 6).

Our new data confirms the importance of IFNγ for development of IH following endoluminal injury, and indicates that it is dependent on IFNγ expression by a BM-derived cell. Having previously shown in this model that BM-derived myofibrocytes influenced the development of IH (8, 9), we subsequently described that adoptively transferred CD34+ cells contained myofibrocytes that were recruited early to the site of injury and influenced neointima formation (14). By day 5, a significant proportion (~75%) of newly recruited collagen-1+ cells (fibrocytes) co-expressed IFNγ, but a similar proportion of F4/80+ cells (macrophages) also co-expressed IFNγ. Together, these two cell types accounted for ~85% of IFNγ-expressing neointimal cells at this early stage post-injury. From these new data, we conclude that IFNγ expression by CD34+ cells, predominantly fibrocytes and macrophages, recruited to the site of injury is both sufficient and necessary to initiate progressive IH.

Our previous work has focused on defining cellular mechanisms through which TF induces IH. In summary, our published data suggests that TF with FVIIa and FX, primarily through thrombin generation, upregulates myofibrocyte secretion of angiopoietin-2 which then directly induces TIE-2-dependent proliferation, reduces spontaneous apoptosis and induces CXCL-12 production. Using the adoptive transfer approach, we have previously shown that all these components were relevant *in vivo*.

In this new work we have attempted to link these previous findings to IFNγ. Our data confirms that early neointimal fibrocytes and macrophages were both TF positive, and all IFNγ-expressing cells co-expressed TF. Moreover, IFNγ expression and secretion by each was dependent on their expression of TF, as it was inhibited by an anti-TF antibody administered at the time of injury, and was not seen in CD31-TFPI-Tg mice, unless these received an inhibitory anti-TFPI antibody at the time of injury.

TF-dependent IFNγ expression and secretion *in vitro* could be induced by FX and FVIIa, but was enhanced by presence of prothrombin. In both cases, IFNγ was inhibited almost completely by a PAR-1 antagonist, and partly by a PAR-2 antagonist, consistent with data showing dose and time-dependent IFNγ secretion induced by a PAR-1 and less so by a PAR-2 agonist. When prothrombin was present, the predominant cell type expressing IFNγ *in vitro* was collagen-1 but not F4/80-positive, but this may reflect the propensity of the former to proliferate *in vitro*.

*In vivo*, there were roughly equal proportions of collagen-1+ IFNγ+ and F4/80+ IFNγ positive neointimal cells present in the neointima by day 5. In our adoptive transfer model, virtually all of these were derived from the adoptively transferred CD34+ cells. Interestingly, these cells, isolated from donor mice 3 days post injury, were all IFNγ-negative at the time of isolation and transfer, suggesting that TF-dependent IFNγ was induced at the point of, or after recruitment to the site of injury. We speculate this may be because the early platelet deposition post-injury (14, 28, 29) facilitates thrombin generation, but have not interrogated these data further to address this point.

Having previously shown that angiopoietin-2 production was important for TF-dependent IH, we here show that angiopoietin-2 is necessary for IFNγ expression. Adoptive transfer of CD34+ cells pre-treated with either an anti-TIE-2 antibody or with siRNA targeted to angiopoietin-2 abolished IFNγ expression (and development of IH (14)) without influencing the recruitment of the adoptively transferred cells and was corroborated by *in vitro* data showing that angiopoietin-2 was a vital intermediate in the thrombin-induced upregulation of IFNγ. Data showing that neointimal cells recruited to the site of injury in IFNγ-deficient mice, which did not develop IH, retained TF and angiopoietin-2 expression confirmed that neither was capable of inducing IH unless IFNγ could be expressed.

Angiopoietin-2 is known to induce or augment cytokine and chemokine production by myeloid cells, including IL-8 (30), IL-6 (30, 31) and various chemokines (14, 31), but we believe this is the first time that it has been shown to induce IFNγ.

In summary, we have provided an analysis of how early myeloid recruits at the site of endoluminal vascular injury express IFNγ, via expression of TF, generation of both FXa and thrombin, signalling through PAR (predominantly PAR-1) and secretion of angiopoietin, which via signalling through TIE-2, induces IFNγ which is critical then for promoting the progressive expansion of intimal cells. These data enhance our previous work in this area and provide novel insights into the pathophysiology of IH, suggesting new translational targets for investigation and potential exploitation in human disease.

## Data availability statement

The raw data supporting the conclusions of this article will be made available by the authors, without undue reservation.

## Ethics statement

The animal study was approved by United Kingdom Home Office PPL 70-8888. The study was conducted in accordance with the local legislation and institutional requirements.

## Author contributions

DC: Data curation, Formal analysis, Methodology, Writing – review & editing. KL: Data curation, Formal analysis, Methodology, Supervision, Writing – review & editing. LW: Data curation, Formal analysis, Writing – review & editing. NM: Data curation, Formal analysis, Writing – review & editing. JM: Conceptualization, Methodology, Writing – review & editing. AD: Conceptualization, Funding acquisition, Supervision, Writing – original draft, Writing – review & editing.

## Supplementary material

Supplementary Figure 1 Co-expression of collagen-1 and F4/80 by day 5 neointimal cells. **(A)** Immunohistology of representative sections through injured mouse carotid arteries harvested on day 5 post-injury. All Sections stained with DAPI (4,6 diamidino-2-phenylindole) nuclear stain (blue) and (red) anti-F4/80 plus (green) anti-collagen-1 as indicated. The annotated white line defines the junction between neointima and media. **(B)** Quantitative analysis of the expression of the markers shown in A, expressed as the proportion of the intimal area occupied by cells expressing a particular marker(s) as indicted in each panel. Graphs show box plots with median and interquartile range (IQR) with whiskers showing upper and lower limits and outliers indicated as single data points. Means are represented with ‘x’. Data derived from mean values from 3 random sections taken from 6 different vessels. Measurements taken by an investigator blinded to the identity of the sections.
