# Supplementary figures and images for: Neointimal hyperplasia after endoluminal injury in mice is dependent on tissue factor- and angiopoietin-2 dependent interferon gamma production by fibrocytes and macrophages
Source: Front Immunol. 2024 Jun 7;15:1345199. doi: 10.3389/fimmu.2024.1345199 (PMC11190261; doi:10.3389/fimmu.2024.1345199)

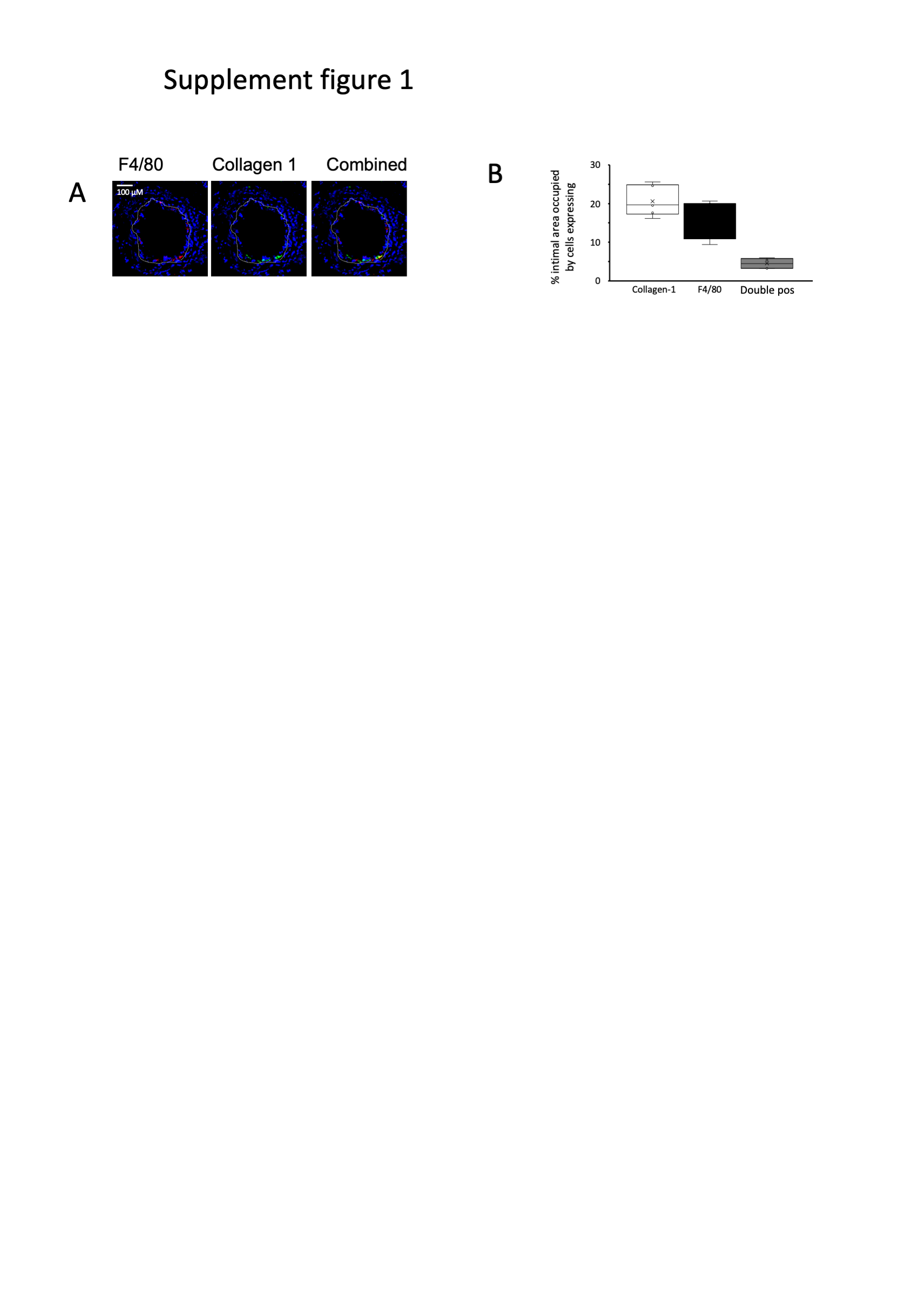

Supplement: Supplementary Figure 1 — Co-expression of collagen-1 and F4/80 by day 5 neointimal cells. (A) Immunohistology of representative sections through injured mouse carotid arteries harvested on day 5 post-injury. All Sections stained with DAPI (4,6 diamidino-2-phenylindole) nuclear stain (blue) and (red) anti-F4/80 plus (green) anti-collagen-1 as indicated. The annotated white line defines the junction between neointima and media. (B) Quantitative analysis of the expression of the markers shown in A, expressed as the proportion of the intimal area occupied by cells expressing a particular marker(s) as indicted in each panel. Graphs show box plots with median and interquartile range (IQR) with whiskers showing upper and lower limits and outliers indicated as single data points. Means are represented with ‘x’. Data derived from mean values from 3 random sections taken from 6 different vessels. Measurements taken by an investigator blinded to the identity of the sections. [file Image_1.tiff]
